# Identification of two novel glial-restricted cell populations in the embryonic telencephalon arising from unique origins

**DOI:** 10.1186/1471-213X-7-33

**Published:** 2007-04-17

**Authors:** Frederick G Strathmann, Xi Wang, Margot Mayer-Pröschel

**Affiliations:** 1 Department of Pathology and Laboratory Medicine, University of Rochester, Rochester, New York 14642, USA; 2 Department of Biomedical Genetics, University of Rochester, Rochester, New York 14642, USA

## Abstract

**Background:**

Considerably less attention has been given to understanding the cellular components of gliogenesis in the telencephalon when compared to neuronogenesis, despite the necessity of normal glial cell formation for neurological function. Early proposals of exclusive ventral oligodendrocyte precursor cell (OPC) generation have been challenged recently with studies revealing the potential of the dorsal telencephalon to also generate oligodendrocytes. The identification of OPCs generated from multiple regions of the developing telencephalon, together with the need of the embryonic telencephalon to provide precursor cells for oligodendrocytes as well as astrocytes in ventral and dorsal areas, raises questions concerning the identity of the precursor cell populations capable of generating macroglial subtypes during multiple developmental windows and in differing locations.

**Results:**

We have identified progenitor populations in the ventral and dorsal telencephalon restricted to the generation of astrocytes and oligodendrocytes. We further demonstrate that the dorsal glial progenitor cells can be generated *de novo *from the dorsal telencephalon and we demonstrate their capacity for *in vivo *production of both myelin-forming oligodendrocytes and astrocytes upon transplantation.

**Conclusion:**

Based on our results we offer a unifying model of telencephalic gliogenesis, with the generation of both oligodendrocytes and astrocytes from spatially separate, but functionally similar, glial restricted populations at different developmental times in the dorsal and ventral CNS.

## Background

Within the central nervous system (CNS), the greatest progress in identifying the specific cell populations involved in development has been achieved in the spinal cord. In the rat spinal cord, E10.5 cells have been shown to represent a homogenous population of multipotent neuroepithelial stem cells (NEPs) capable of generating cells of both the neuronal and glial lineage. Differentiated cell types arise from these NEP cells by way of lineage restricted intermediate precursor populations capable of extended proliferation and the generation of neurons or glia. The cells comprising the earliest intermediate precursor population restricted to oligodendrocyte and astrocyte formation, called glial restricted precursor cells (GRPs), can be isolated from the embryonic spinal cord as early as E12. Their ability to generate two antigenically distinct populations of astrocytes and oligodendrocytes has been established both *in vitro *and *in vivo *(for review see [1,2]). GRP cells are identified with the A2B5 antibody and do not express the Polysialylated form of Neural Cell Adhesion Molecule (PSA-NCAM). Freshly isolated GRP cells depend on basic fibroblast growth factor (bFGF) for survival and proliferation but, unlike oligodendrocyte progenitor cells (OPCs [3]), are not defined by the expression of platelet-derived growth factor receptor-alpha (PDGFR-alpha) or Olig2 [2]. The OPC has been shown *in vivo *to arise at a later time point than the GRP, and the generation of oligodendrocytes from a GRP population has been demonstrated *in vitro *to occur through an OPC intermediate stage [4]. Importantly, in both the GRP and OPC populations, the term restricted is used to underscore the greatly diminished, if not non-existent, capacity for neuronal generation when compared to multipotent NEP cells. To date, GRP cells isolated from the spinal cord have failed to generate neurons in numerous paradigms including transplantation into the embryonic spinal cord [5-7]. It has, however, been reported that glial precursor cells isolated from the postnatal optic nerve can be induced to express neuron-like features if cultured for at least one month in serum containing medium [8], although the significance of this "neuronal potential" remains unclear.

Additional characteristics distinguishing GRP cells from OPCs are the ability of GRP cells to generate two types of astrocytes (that have been designated type-1 and type-2 [7]) *in vitro *and to generate both oligodendrocytes and astrocytes *in vivo*. Both type-1 and type-2 astrocytes are GFAP+, but only type-1 astrocytes co-label with the A2B5 antibody. Type-1 astrocytes are thought to arise from GRP cells through intermediate astrocyte progenitor cells (APC) [9], while type-2 astrocytes may require prior generation of OPCs as an intermediate step [4]. Unlike OPCs, GRP cells readily generate astrocytes following transplantation into the adult CNS [10], while primary OPCs thus far only generate oligodendrocytes in such transplantations [11].

The identification of GRP cells in the spinal cord gave rise to a generalized model of gliogenesis consistent with the majority of experimental data available. This model of gliogenesis involves the progression from a multipotential NEP cell to a lineage restricted multipotent precursor cell population (e.g. GRPs) that in turn give rise to more restricted glial precursor cell types (e.g. OPCs and possibly APCs) and the eventual mature glial cells of the CNS (e.g. oligodendrocytes and astrocytes)[2,12-14]. While the generation of each cell type in the lineage and the resultant appropriate cellular fate of astrocyte or oligodendrocyte are governed by environmental cues, not all potential cell fates that are observed *in vitro *may be witnessed *in vivo*, requiring a careful consideration when interpreting *in vitro *and *in vivo *experiments.

In contrast to the spinal cord, the identification of intermediate cellular components of glial cell generation in the telencephalon is largely incomplete. With the OPC the major focus thus far in studies on glial cell generation in the telencephalon, the extent of similarity between intermediate glial precursor cells of the spinal cord and telencephalon is largely unknown. It has been ascertained through genetic and clonal *in vitro *experiments that a subset of cells from ventral regions of the telencephalon differentiate into PDGFR-alpha+ and/or Olig2+ oligodendrocyte progenitors, migrate away from their ventral origin, and give rise to mature oligodendrocytes throughout the brain [15-20]. It is further assumed that these cells need to express Olig1/2 to be fated towards oligodendrocytes as compound disruption of *Olig1 *and *Olig2 *results in a complete loss of oligodendrocytes [21-27]. While these experiments led to the view that the major source for telencephalic oligodendrocytes are ventrally generated OPCs, recent evidence indicates there might be a dorsal origin for a subset, if not majority, of telencephalic oligodendrocytes [27].

Several populations of PDGFR-alpha+ OPCs in the telencephalon have been identified, each with distinctive spatial and temporal origins [24,26,27], but whether the OPC represents the only glial restricted cell in the telencephalon remains unknown. This deficit in our understanding of the glial progenitor populations present in the developing telencephalon also raises the question as to which cells are involved in the generation of astrocytes, a critical cell component of the telencephalon. In addition, while it is well established that cortical NEPs generate neurons, astrocytes, and oligodendrocytes, it is unclear whether or not mature, cortical glial cells are derived from lineage restricted precursor cells or are the product of migrating stem cells *in vivo*. Although precursor cell populations responsible for glial cell formation in the telencephalon have been described [20,24,26,28], to date no embryonic telencephalic cell has been identified and isolated that possesses the ability to generate both oligodendrocytes and astrocytes in the absence of neuron generation *in vitro *or *in vivo*.

With a growing interest in and an increased appreciation for the therapeutic potential of spinal cord derived GRPs [29] and the role of precursor populations in disease [30], the aims of this study were to investigate the presence of progenitor populations capable of generating oligodendrocytes and astrocytes but unable to generate neurons, and to determine whether such a progenitor population is derived dorsally and/or ventrally. We began our analysis by isolating cell populations from the dorsal telencephalon based on the antigenic phenotype of restricted precursor cells previously identified in the spinal cord. These telencephalic cells were characterized in mass culture and at the clonal level and were found to generate all macroglial subtypes but were unable to generate neurons. We further determined the dorsal telencephalon is capable of generating this glial restricted population *de novo *by separating the dorsal telencephalon at a time point where the cell populations present are exclusively of a dorsal origin. In line with the potential dorsal origin of this glial restricted cell population, we identified a ventral glial restricted cell population in parallel. We confirmed the ability of the dorsal cell population to differentiate into myelin producing oligodendrocytes upon transplantation in a myelin deficient background, as well as GFAP+ astrocytes when transplanted into the perinatal forebrain. To our knowledge, these findings represent the first identification of progenitor cells in the embryonic telencephalon that are able to generate both oligodendrocytes and astrocytes but are unable to generate neuronal progeny. Our study also provide for the first time a defined cell population that is generated *de novo *in the dorsal aspect of the telencephalon and could be the source for both dorsally derived oligodendrocytes and astrocytes. Taken together, our findings provide a general model of gliogenesis by which glial cells originate in a timely and organized manner in the developing telencephalon. This identification and characterization of a telencephalic glial restricted progenitor population is an important step in understanding early telencephalic oligodendrocyte and astrocyte generation and provides a foundation for further investigation into normal and abnormal telencephalic glial cell development.

## Results

### A2B5+ cells can be detected in the dorsal telencephalon outside of the ventral Olig2 domain

We chose the dorsal telencephalon to pursue our initial identification of a glial restricted progenitor in the telencephalon as this region provides two major advantages over the ventral telencephalon for cell identification: First, OPCs are not detected in the dorsal telencephalon until after E15 (based on PDGFR-alpha expression [21]), while the ventral telencephalon has been reported to contain OPCs (defined as PDGFR-alpha+ cells) as early as E12.5 [31]. As both GRPs and OPCs are A2B5+ [3,7], an initial distinction between these two cell types necessitated cell isolation from a specific developmental window in a region such as the E15 dorsal telencephalon, known to possess gliogenic potential but being devoid of the OPC [15,32]. Second, the dorsal telencephalon consists entirely of dorsal born cells until the time of ventral cell infiltration, at approximately E13.5 in the rat [33], providing the opportunity to explore the origin of an identified progenitor population.

**Figure 1:**
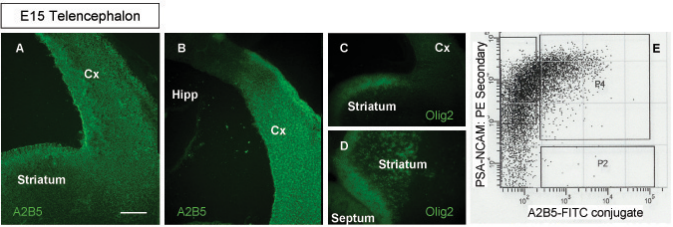
**A2B5+ cells in the telencephalon**. (A), A2B5+ cells are seen in coronal sections of the developing striatum and dorsolateral neocortex of the E15 telencephalon. (B) A2B5+ cells are absent in the developing hippocampal region. (C,D) The dorsal A2B5+ region is not Olig2+ (C) while the ventral A2B5+ region partially overlaps with the Olig2+ domain in the developing striatum (D). (E) FACS data of A2B5+/PSA-NCAM- stained cells shows three cell populations, including PSA-NCAM+, A2B5+/PSA-NCAM+, and A2B5+. Scale bar, 100 μm.

We first characterized the distribution of A2B5+ cells in the embryonic telencephalon, and as shown in Figure 1A and 1B, A2B5 labeled cells are present in both the E15 dorsal and ventral telencephalon, whereas Olig2, a marker for OPCs, was found only in the ventral telencephalon (Fig. 1C and 1D), consistent with previous reports [31]. Due to the difficulties inherent in obtaining single cell identification with the A2B5 stained sections, we were unable to resolve with any greater specificity the precise region or regions of the dorsal and ventral telencephalon where A2B5 labeling was likely to be attributed to putative glial restricted progenitor cells, maturing neurons, or other neural populations. To determine the presence of a glial restricted progenitor population among the widely A2B5 positive telencephalon, cell isolation and sorting was conducted using the antigenic phenotype that defines spinal cord GRP cells: A2B5+/PSA-NCAM- [5,7]. As A2B5 and anti-PSA-NCAM are both IgM antibodies, we used an A2B5 primary antibody directly conjugated to fluorescein allowing for simultaneous labeling of A2B5 and anti-PSA-NCAM immunoreactive cells. FACS analysis revealed three distinct cell populations: PSA-NCAM+ only cells, A2B5+ only cells, and cells that co-label with anti-PSA-NCAM and A2B5 (Fig. 1E). These results confirm the presence of an A2B5+/PSA-NCAM- cell population in the dorsal telencephalon located outside of the Olig2 domain. The A2B5+ only population was the focus of further analysis as this antigenic phenotype is shared by the previously identified spinal cord GRP cell [7]. It is important to note, however, that both the A2B5+/PSA-NCAM+ and the PSA-NCAM+ only populations contained at least a subset of cells capable of glial cell generation, as seen in preliminary mass culture experiments (data not shown).

### A2B5 labels a subset of neurons in the dorsal telencephalon

**Figure 2:**
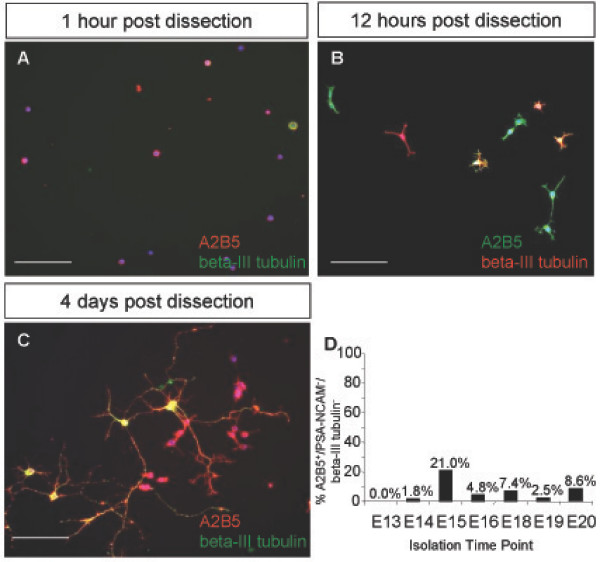
**A subset of A2B5+ cells are also beta-III tubulin+ in the E15 dorsal telencephalon**. (A-C) The isolated A2B5+/PSA-NCAM- cell population from the dorsal telencephalon included a beta-III tubulin+ population, seen at 1 hour (A), 12 hours (B), and 4 days (C) post isolation. (D) Isolated A2B5+/PSA-NCAM- cells were stained and analyzed for beta-III tubulin presence between E13 and E20. E15 was determined to be the peak time to isolate A2B5+/PSA-NCAM-/beta-III tubulin- cells as 22% of the E15 A2B5+/PSA-NCAM- population was beta-III tubulin-. DAPI, blue nuclear stain. Scale bars, 100 μm.

The purification of A2B5+/PSA-NCAM- cells from the E15 dorsal telencephalon yielded a heterogeneous population of putative glial progenitors and neurons. A2B5+/PSA-NCAM- populations isolated as early as E13 to as late as E20 from the dorsal telencephalon contained A2B5+ cells expressing the neuronal marker beta-III tubulin, detected by immunofluorescence at 4 hours, 12 hours and 4 days post-dissection (Fig. 2A–C). The lack of glial precursor-restricted labeling with A2B5 prompted us to examine the A2B5+/PSA-NCAM- cell populations in combination with beta-III tubulin to determine the appropriate developmental time point that would yield specifically A2B5+/PSA-NCAM-/beta-III tubulin- cells. Acute staining of cells directly after dissection indicated that the peak time for isolating an optimal number of A2B5+/PSA-NCAM-/beta-III tubulin- cells was E15, when A2B5+/beta-III tubulin- cells represented approximately 22% of the subpopulation of A2B5+/PSA-NCAM- E15 dorsal telencephalic cells (Fig. 2D). In addition, BrdU injection of the pregnant females 4 hours prior to dissection identified the isolated A2B5+ neuronal population as postmitotic (BrdU negative, data not shown). We therefore used E15 as the peak time point to isolate a putative glial restricted progenitor population identified as A2B5+/PSA-NCAM-/beta-III tubulin-; however, the overlap of A2B5 immunoreactivity on neuronal as well as glial cells confounded a simple separation of the ventral and dorsal putative glial precursor populations from the A2B5+ neuronal populations.

As we could not identify a neuron specific cell surface marker that would allow the separation of the neuronal population (A2B5+/PSA-NCAM-/beta-III tubulin+) from the remaining progenitor population (A2B5+/PSA-NCAM-/beta-III tubulin-), an alternative approach was taken, discussed at length in the Materials and Methods section, using observed differences in adhesion and survival between the neuronal population and the progenitor population.

### Defining the A2B5+/PSA-NCAM-/beta-III tubulin- population

To further characterize the antigenic profile of the A2B5+/PSA-NCAM-/beta-III tubulin- putative glial restricted progenitor population, freshly isolated and MACS sorted cells were allowed to adhere to a FN/LN coated surface over a maximum of 8 hours. Cells were then stained with antibodies directed against spatially relevant and cell-type specific antigens. Table 1 provides a summary of the antibodies used and the determined presence or absence of their respective antigens in the putative glial restricted progenitor population. More mature glial markers were absent as expected, including Olig2, PDGFR alpha, NG2, GFAP, CD44, S100, SOX10, Ran2 and O4. Antigens associated with neurons and their progenitors including NeuN and Doublecortin were not detected. Cells were also negative for the radial glial marker 3CB2.

**Table 1:** Antigenic profile of the A2B5+/PSA-NCAM- population, pre- and post-*in vitro *growth

Antigen	Freshly isolated A2B5+/PSA-NCAM- cells	*In vitro *expanded A2B5+/PSA-NCAM- cells
A2B5	+	+
CD44	-	-
GFAP	-	-
Nestin	+	+
NG2	-	-
O4	-	-
Olig2	-	-
PDGFR alpha	-	-
PSA-NCAM	-	-
Ran2	-	-
S100	-	-
3CB2	-	-
Sox2	+	+
Sox10	-	-
Beta III Tubulin	+	-

In contrast to the absence of neuronal markers and more mature glial lineage markers, putative glial restricted progenitor population were immunoreactive for both Nestin and Sox2, antigens that have been shown to be present in various populations of stem cells [35-38], GRP cells [1,7,39], and proliferating astrocytes [37]. While the antigenic profile of the A2B5+/PSA-NCAM-/beta-III tubulin- cell population was not consistent with OPCs, the expression of Nestin and Sox2 did not allow us to distinguish between stem cells and GRP cells. As stem cells differ from GRP cells in their differentiation potential *in vitro *and *in vivo*, we conducted a number of experiments that were geared towards the identification of the differentiation potential of the A2B5+/PSA-NCAM-/beta-III tubulin- cell population. To determine a possible lineage restriction of the A2B5+/PSA-NCAM- cell population, it was essential to culture the defined cell population over a minimum of 7 days in a defined condition that allows the expansion of the cells without changing their phenotype.

To establish such a condition, freshly isolated, MACS sorted A2B5+/PSA-NCAM- cells (comprised of a heterogeneous population of A2B5+/PSA-NCAM-/beta-III tubulin+ and of A2B5+/PSA-NCAM-/beta-III tubulin-) were plated in defined medium supplement with bFGF and cultured for 7 days. To determine whether the cells remained unchanged during *in vitro *growth, the resultant population that was grown for 7 days as describe above and passaged twice were stained with the antibodies listed in Table 1 and compared to freshly isolated cells. The antigenic profile of the cell population that underwent growth and expansion in bFGF *in vitro *was identical to the antigenic profile of freshly isolated and MACS sorted cells, with the exception of the loss of beta-III tubulin detection (see Table 1). Importantly, the A2B5+/PSA-NCAM-/beta-III tubulin- cell population remained Olig2 negative (even after 3 weeks of *in vitro *growth in basal media supplemented with 10 ng/ml bFGF (data not shown)). This observation is important as it has been suggested by Gabay et al that bFGF might have a "ventralizing" effect on Olig2 negative dorsal derived spinal cord cells [40]. Our results did not suggest such a role of bFGF in the dorsal-derived telencephalic A2B5+/PSA-NCAM-/beta-III tubulin- cells. In addition, we did not see any spontaneously appearing beta-III tubulin+ cells or any obvious differences in cell morphology, growth rate, or survival during this *in vitro *growth, further arguing against the "ventralizing" effects in response to bFGF as described by Gabay et al., 2003.

### The A2B5+/PSA-NCAM- population generates astrocytes and oligodendrocytes in mass culture but does not generate neurons

**Figure 3:**
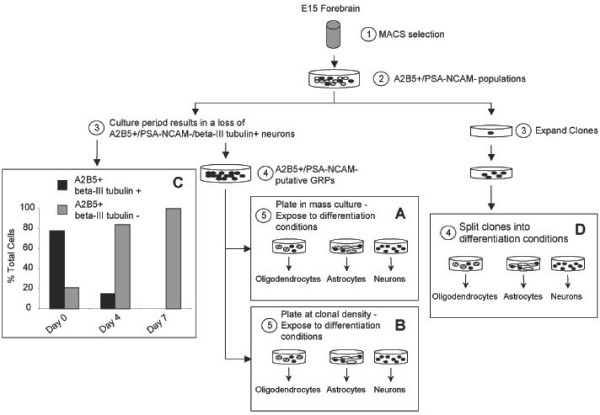
**Outline of the isolation procedure used to characterize the putative glial restricted progenitor population**. A2B5+/PSA-NCAM- cells were selected by MACS resulting in a heterogeneous mixture of cells. For mass culture studies (A) and clonal analysis (B), cells were maintained in culture for two cell passages to select for proliferative cells and remove the A2B5+ neuronal population (C). The resultant putative glial restricted progenitor population was then plated at mass culture or clonal density and exposed to differentiating conditions including a pro-oligodendrocytic condition, a pro-astrocytic condition, or a pro-neuronal condition (see Materials and Methods). Alternatively, the heterogeneous mixture of cells obtained from the MACS selection was plated at clonal density, and resultant clones were selectively passaged and split into the differentiation conditions (D).

The culture conditions we identified allowed for the expansion of cells while maintaining their antigenic phenotype. We used this *in vitro *culture system to determine whether the A2B5+/PSA-NCAM-/beta-III tubulin- population represented neural stem cells or lineage restricted precursor cells. While both cells population share a similar antigenic profile, their *in vitro *and *in vivo *differentiation potential would be fundamentally different. Neural stem cells are considered to be multipotent and are able to give rise to glial as well as neuronal populations. In contrast, lineage restricted cells have lost their multipotency and are restricted in their differentiation potential to either glial or neuronal lineages or to a specific subset of cells of either lineage. To determine the differentiation potential of the A2B5+/PSA-NCAM-/beta-III tubulin- cell population from the E15 dorsal telencephalon, we conducted mass culture analyses (as shown in Figures 3A), clonal analyses (3B), and clonal splitting analyses (3D). Each experiment was designed to determine the ability of the isolated cell populations to generate astrocytes, oligodendrocytes and neurons. Differentiation conditions used for these analyses were based on our previous data on spinal cord derived GRPs [4,7,10] and on many reports in the literature. As a pro-astrocyte condition, cells were exposed to 2% FBS. To determine whether cells are capable of generating oligodendrocytes, cultures were exposed to PDGF-AA plus T3/T4 (pro-oligodendrocyte). To facilitate neuronal differentiation cells, were exposed to NT3 plus RA (pro-neuron), a condition that has been shown to be effective in directing beta-III tubulin+ neuron formation from spinal cord NEP cells [41]. Control cultures were kept in bFGF and represented the proliferate condition.

**Figure 4:**
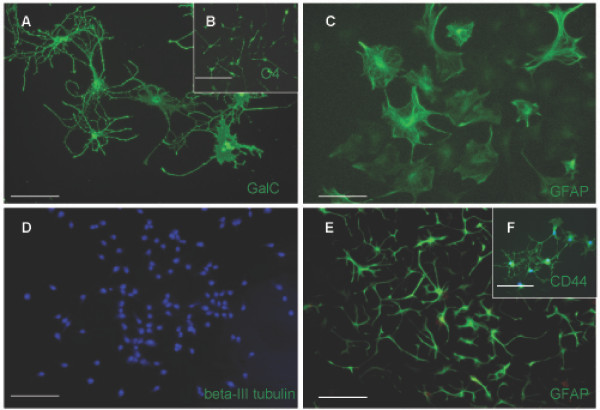
**The putative dorsal glial restricted progenitor population can generate macroglial subtypes in mass culture**. (A,C,D) Putative glial restricted progenitor cells generate GalC+ cells (A) GFAP+ cells (C) but do not generate neurons (D) after 6 days of exposure to the appropriate differentiation conditions (see Results). (B) After 4 days of growth in the pro-oligodendrocyte condition, O4+ cells were readily identifiable. (E,F) Exposure of the putative glial restricted progenitor population to BMP-4 is insufficient to result in detection of the known astrocyte marker GFAP until 10 days (E), but does induce the Astrocyte Precursor Cell marker, CD44, after 6 days (F). DAPI, blue nuclear stain (D,F). Scale bars, 100 μm.

**Figure 5:**
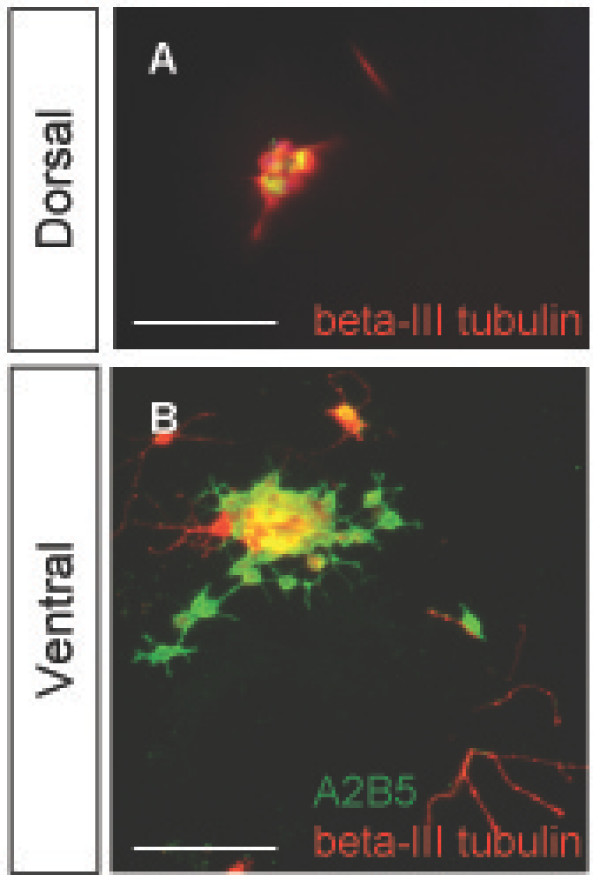
**Neuron generation from E15 unsorted dorsal and ventral telencephalic cells**. In order to validate the pro-neuronal condition used, cells present in the E15 dorsal (A) and ventral (B) telencephalon before MACS selection were exposed to the pro-neuronal condition used for glial restricted progenitor characterization and were found to generate beta-III tubulin+ cells after 6 days in culture. Scale bars, 100 μm.

Cells were isolated from the E15 dorsal telencephalon, MACS sorted for A2B5+/PSA-NCAM- cells and expanded for 7 days in bFGF. Cultures were then switched to differentiation conditions and labeled after 6–9 days (depending on condition) with markers that identified differentiated progeny. As show in Figure 4A,C and 4D, cells were capable of generating GalC+ oligodendrocytes in PDGF-AA plus T3/T4 and GFAP+ astrocytes in 2% FBS, but were unable to generate neurons in NT3 and RA. To exclude the possibility that the failure of neuronal generation from the A2B5+/PSA-NCAM-/beta-III tubulin- was due to an inadequate pro-neuronal environment, we cultured freshly isolated, non-selected cells from E15 dorsal telencephala at clonal density in the presence of NT3 and RA for 6 days and labeled clones with anti-beta-III tubulin. As shown in Figure 5A, clones possessing the ability to generate neurons in the pro-neuron condition were readily identifiable, indicating the pro-neuronal condition used was adequate to elicit neuron formation from a competent cell.

In accordance with the generation of oligodendrocytes from spinal cord derived GRP cells, an O4+ intermediate cell type was seen upon exposure to PDGF-AA plus T3/T4 for 4 days (Fig. 4B)[4] Interestingly, BMP-4, shown previously to increase astroglial cell commitment [42] and implicated in the switch from neuron to astrocyte formation in the telencephalon [43] was unable to generate GFAP+ cells until 10 days after the onset of BMP exposure (Fig. 4E), but did induce expression of the known GRP derived astrocyte progenitor cell marker, CD44 [9], after 6 days *in vitro *(Fig. 4F). Taken together, the results presented thus far confirmed that the A2B5+/PSA-NCAM- dorsal telencephalic cell population is capable of generating oligodendrocytes and astrocytes but not neurons.

### The A2B5+/PSA-NCAM- population generates similar numbers of clones containing oligodendrocytes or astrocytes, but no clones containing neurons

While the initial *in vitro *differentiation experiments indicated the restriction of the A2B5+/PSA-NCAM- population to the glial lineage, a distinction between the presence of a bipotential cell that can generate oligodendrocytes and astrocytes and the presence of a heterogeneous population of APCs and OPCs was necessary. To distinguish between these two possibilities, A2B5+/PSA-NCAM- cells grown in culture for one week were passaged and re-plated at clonal density. Clones were then exposed to bFGF (proliferative), PDGF-AA plus T3T4 (pro-oligodendrocyte), 2% FBS (pro-astrocyte), or NT3 plus RA (pro-neuron) in order to determine the differentiation potential of individual clones. A clone was considered to be capable of generating the specified cell types by the presence of at least one oligodendrocyte per clone, at least one astrocyte per clone, or at least one neuron per clone, in the respective condition.

**Figure 6:**
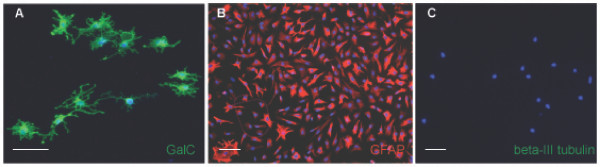
**Clonal analysis of the putative dorsal glial restricted progenitor further indicates glial restriction**. (A-C) To distinguish between the potential presence of an APC/OPC cell mixture and the presence of a glial restricted progenitor population, the putative glial restricted progenitor population was grown at clonal density and exposed to the differentiating conditions, resulting in the detection of clones containing GalC+ cells (A) clones containing GFAP+ cells (B) but no neuron containing clones (C). DAPI blue nuclear stain. Scale bars, 100 μm.

**Figure 7:**
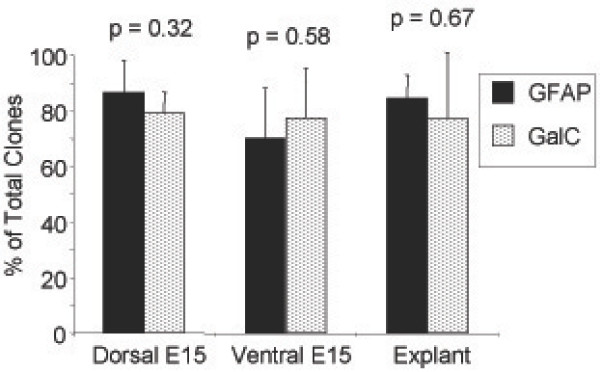
**Clonal Summary**. A summary of the generated clones from Dorsal, Ventral, and Explant derived glial restricted progenitor is provided, with no significant difference (p > 0.05; Student's t-test) between astrocyte and oligodendrocyte containing clone numbers.

A2B5+/PSA-NCAM- cells from the dorsal telencephalon gave rise to clones capable of generating oligodendrocytes (Fig. 6A), astrocytes (Fig. 6B) but not neurons (Fig. 6C) after six days of exposure to the differentiation conditions. In four independent experiments, a total of 223 clones exposed to PDGF-AA plus T3/T4, a total of 164 clones exposed to 2% FBS, and more than 200 clones exposed to NT3 plus RA were analyzed. 79% of the clones exposed to PDGF-AA plus T3/T4 contained at least one GalC+ oligodendrocyte, 87% of all clones exposed to 2% serum (115 clones) contained at least one GFAP+ astrocyte, while none of the clones exposed to NT3 plus RA contained a neuron. A summary of the GFAP+ and GalC+ clones is presented in Figure 7, and indicates a similar percentage of astrocyte-containing clones and oligodendrocyte-containing clones in the respective conditions, a result consistent with a cell capable of generating both oligodendrocytes and astrocytes.

### The splitting of A2B5+/PSA-NCAM- clones reveals the potential to generate oligodendrocytes and astrocytes from a single founder cell

**Figure 8:**
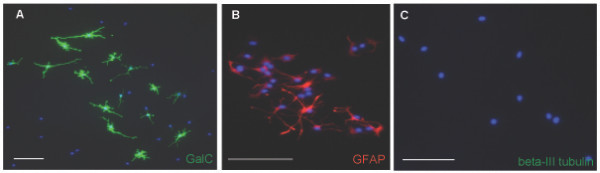
**Clone splitting confirms the ability of the putative glial restricted progenitor cell to generate both oligodendrocytes and astrocytes**. Split clones (see Results) of A2B5+/PSA-NCAM- founder cells can generate GalC+ cells (A) GFAP+ cells (B) but not neurons (C) and allows for the classification of the A2B5+/PSA-NCAM-/beta-III tubulin- cell as a glial restricted progenitor cell. DAPI, blue nuclear stain. Scale bars, 100 μm.

The analysis of the clonal data strongly suggested the A2B5+/PSA-NCAM- population consisted of a cell capable of generating both oligodendrocytes and astrocytes when exposed to appropriate conditions in parallel wells. As the presently known conditions that are required to induce cell differentiation along a specific lineage do not allow the generation of oligodendrocytes and astrocytes in a single clone at the same time, an alternative method was needed to determine whether the progeny arising from a single A2B5+/PSA-NCAM- cell was able to generate oligodendrocytes and astrocytes. We therefore initiated a "clone-splitting" analysis, as outlined in Figure 3D. The cells were plated at clonal density in 100 mm dishes and allowed to propagate in bFGF (10 ng/ml) until a clone size of approximately 200 cells was achieved. Clones were selected based on the presence of cells consistent with the bipolar morphology of progenitor cells. Each selected clone was passaged and re-plated amongst four wells of a 24 well plate and exposed to the previously used differentiating conditions. Clones passaged in this manner gave rise to oligodendrocytes in PDGF-AA plus T3T4 (Fig. 8A), astrocytes in 2% FBS (Fig. 8B) but did not generate neurons in NT3 and RA (Fig. 8C) after 6 days of exposure to the indicated conditions. Each split clone was capable of generating oligodendrocytes and astrocytes but not neurons in the respective conditions, confirming the potential of the initial A2B5+/PSA-NCAM- founder cell to generate both oligodendrocytes and astrocytes, and allowing for its classification as a glial restricted progenitor cell.

### Dorsal glial restricted progenitor cells are generated *de novo *from the dorsal telencephalon

The identification of a glial restricted progenitor cell population from the dorsal telencephalon raises the question as to the origin of this population *in vivo*. In order to determine if the dorsal telencephalon is competent to generate glial restricted progenitor cells *de novo*, or is a result of ventral cell infiltration, we mechanically separated the E12.5 dorsal telencephalon from the ventral telencephalon and grew the dorsal explant for 2 days *in vitro*. The physical separation of the dorsal telencephalon from the ventral telencephalon allowed for the simulated development of the dorsal telencephalon in the absence of ventral cell types until a time period comparable to an E15 dorsal telencephalon. As E12.5 is prior to the known entrance of ventral cells into the dorsal telencephalon [33], any cells present or generated in the two day culture period were decisively of dorsal origin.

Explants were harvested after two days of *in vitro *growth in Neural Basal Media in the absence of bFGF. This was important as we wanted to minimize the possibility that the culture conditions would lead to a "ventralization" of the explants, although, as described above, we did not observe such an effect *in vitro *when we cultured dissociated cells in the presence of bFGF.

**Figure 9:**
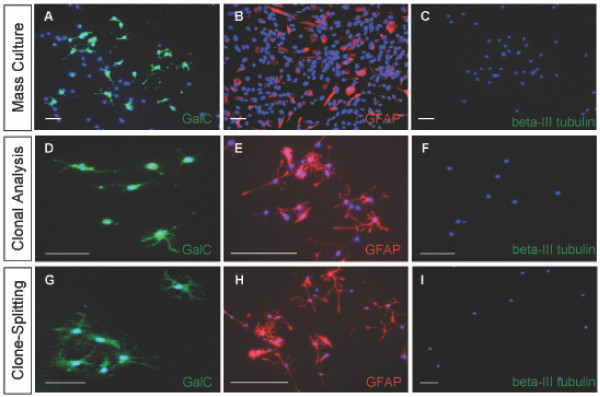
**The dorsal telencephalon has the potential to generate glial restricted progenitor cells independent of ventral cell infiltration**. (A-C) Cells with the similar antigenic profile described for the dorsal glial restricted progenitor population were isolated from two day *in vitro *grown dorsal explants, and can generate GalC+ cells (A) GFAP+ cells (B) but not neurons (C) in mass culture. (D-F) Explant derived putative glial restricted progenitors can generate clones containing GalC+ cells (D) clones containing GFAP+ cells (E) but no clones containing neurons (F) when exposed to the differentiation conditions. (G-I) Split clones of explant derived putative glial restricted progenitor founder cells can generate GalC+ cells (G) GFAP+ cells (H) but not neurons (I). DAPI, blue nuclear stain. Scale bars, 100 μm.

Explant tissue was cultured for 2 days, after which A2B5+/PSA-NCAM- cells were selected by MACS separation from the dissociated explants and cultured for an additional 7 days before being subjected to mass culture differentiation and clonal analyses. Mass culture studies indicated that the explant-derived A2B5+/PSA-NCAM- cell population possessed similar *in vitro *differentiation abilities as the glial restricted progenitor population from the dorsal telencephalon. Explant cells were induced to generate GalC+ oligodendrocytes with PDGF-AA plus T3/T4 (Fig. 9A), GFAP+ astrocytes with 2% FBS (Fig. 9B), and did not generate neurons in NT3 plus RA (Fig. 9C). The explant derived A2B5+/PSA-NCAM- cells grown at clonal density gave rise to 145 out of 190 (76%) clones containing at least one GalC+ oligodendrocyte when exposed to PDGF-AA plus T3/T4 (Fig. 9D). 144 out of 173 (84%) clones contained at least one astrocyte when exposed to 2% FBS (Fig. 9E), and clones containing at least one neuron when exposed to NT3 and RA could not be detected (Fig. 9F). A summary of the clones generated by the dorsal explant A2B5+/PSA-NCAM- cell population is provided (Figure 7).

To further the characterization of the explant derived putative glial restricted progenitor population, A2B5+/PSA-NCAM- cells isolated from 2 day *in vitro *grown explants were plated at clonal density and the differentiation potential of the clonal progeny was characterized as outlined in Figure 3D. Six clones were selectively passaged and the cells from each clone were divided among four wells of a 24 well plate for exposure to the differentiation conditions. Cells from the split clones were able to generate GalC+ oligodendrocytes in PDGF-AA plus T3/T4 (Fig. 9G), GFAP+ astrocytes in 2% FBS (Fig. 9H), but were unable to generate neurons in NT3 and RA (Fig. 9I). These data confirm the ability of the dorsal telencephalon to give rise to an A2B5+/PSA-NCAM- glial restricted progenitor population independent of cellular migration from ventral regions and indicates a potential dorsal origin for the telencephalic glial restricted progenitor population *in vivo*.

### A ventral glial restricted progenitor cell can be isolated from the E15 rat telencephalon

While our experiments show the existence of a novel glial restricted progenitor cell in the dorsal telencephalon, others have suggested that such cells might also exist in the ventral aspect of the developing telencephalon as early as E12.5 in mouse [24,31,44,45]. As no ventral telencephalic cell from the developing telencephalon has been reported to be able to give rise to astrocytes and oligodendrocytes but not neurons, we expanded our analysis and asked whether a glial restricted precursor cell also exists in the ventral aspect of the early telencephalon.

Due to the multiple origins of OPC generation, we began our analysis of a putative ventral glial restricted progenitor population by dissecting the medial ganglionic eminence (MGE) and the anterior entopeduncular area (AEP) of E15 ventral telencephala. *Pdgfr-alpha *expression studies indicated OPC presence in these areas [21], although the cellular origin of the OPCs is not known. The potential problem of isolating a heterogeneous population of glial restricted progenitor cells and OPCs was addressed by growing freshly isolated A2B5+/PSA-NCAM- cells in the presence of 10 ng/ml PDGF. This condition has been previously shown to maintain OPCs but unable to support GRP cell survival [7]. Surviving cells grown in this manner were beta-III tubulin+ and no more than two A2B5+ cells per 1 × 10^6 ^total cells were detected (data not shown). Taken together, the absence of a PDGF responsive A2B5+ population and the known inability of OPCs to generate type-1 astrocytes (A2B5-/GFAP+) allowed for the selective determination of a novel ventral glial restricted population.

**Figure 10:**
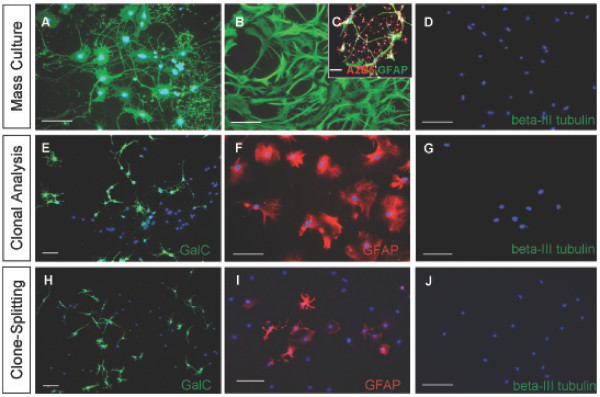
**A glial restricted progenitor population cell can be isolated from the E15 ventral telencephalon**. (A,B,D) Putative glial restricted progenitor cells sharing the similar antigenic profile of the dorsal glial restricted progenitor population were isolated from the E15 ventral telencephalon, consisting of the AEP and MGE. This cell population generated GalC+ cells (A) GFAP+ cells (B) but not neurons (D) in mass culture. (C) Putative glial restricted progenitor cells do not make A2B5+/GFAP+ type-2 astrocytes in response to CNTF. (E-G), To distinguish between APC/OPC presence and glial restricted progenitor presence, ventral putative glial restricted progenitor cells were grown at clonal density and generated GalC+ cells (E) GFAP+ cells (F) but not neurons (G) when examined at the clonal level. (H-J) Split clones of ventral putative glial restricted progenitor founder cells generated GalC+ cells (H) GFAP+ cells (I) but not neurons (J). DAPI, blue nuclear stain, (A,C-J). Scale bars, 100 μm.

A2B5+/PSA-NCAM- cells were isolated and characterized *in vitro *using the same experimental approaches described before and summarized in Figure 3. Mass culture studies confirmed the ability of this ventral A2B5+/PSA-NCAM- cell population to generate GalC+ oligodendrocytes in PDGF-AA plus T3/T4 (Fig. 10A), GFAP+ astrocytes in 2% FBS (Fig. 10B) and the inability to generate neurons in NT3 and RA (Fig. 10D). Clonal analysis established the capacity of individual A2B5+/PSA-NCAM- cells to generate 174 out of 223 (78%) total clones counted containing at least one GalC+ oligodendrocytes in PDGF plus T3T4 (Fig. 10E), 115 clones out of 164 (70%) total clones counted containing at least one GFAP+ astrocytes (Fig. 10F), but an inability to generate clones containing at least one neuron in NT3 and RA (Fig. 10G). A summary of the clones counted is provided in Figure 7. In order to confirm the effectiveness of NT3 and RA to induce a neuronal cell fate, freshly isolated unselected ventral telencephalic cells were plated at clonal density. Unselected cells from the ventral telencephalon possessing the necessary differentiation potential generated beta-III tubulin+ cell clones identifiable after 6 days of exposure to NT3 plus RA (Fig. 5B).

A2B5+/GFAP+ cells were not detected in 2% FBS or with exposure to ciliary neurotrophic factor (CNTF; Fig. 10C), a condition known to induce A2B5+/GFAP+ Type-2 astrocytes from spinal cord derived GRPs [7]. Type-2 astrocyte generation and oligodendrocyte generation is presently thought to be the differentiation profile of the OPC, while the ability to generate both Type-1 (A2B5-/GFAP+) and Type-2 (A2B5+/GFAP+) astrocytes and GalC+ oligodendrocytes from a restricted glial precursor is characteristic only of the GRP cell. The inability to detect Type-2 astrocyte formation from the telencephalic glial restricted progenitor population is likely attributable to as yet undetermined differences between spinal cord GRPs and telencephalic glial restricted progenitor cells.

For further *in vitro *characterization, freshly isolated ventral A2B5+/PSA-NCAM- cells were plated at clonal density and selectively passaged and split as outlined in Figure 3D. The cells from a single divided clone generated GalC+ oligodendrocytes in PDGF-AA plus T3/T4 (Fig. 10H), GFAP+ astrocytes in 2% FBS (Fig. 10I) but did not generate neurons in NT3 plus RA (Fig. 10J). These results confirm glial restricted progenitor cells are present in the E15 ventral telencephalon.

### *In vivo *production of myelinating oligodendrocytes and astrocytes by telencephalic glial restricted progenitor cells

Our *in vitro *analyses identified the existence of dorsal and ventral A2B5+/PSA-NCAM- glial restricted progenitor populations in the E15 telencephalon capable of generating oligodendrocytes and/or astrocytes but unable to generate neurons. Our data also indicate that the dorsal telencephalon possesses the potential to generate the A2B5+/PSA-NCAM- glial restricted progenitor population without the presence of ventral cell components. As recent data from several other laboratories have begun to confirm the ability of the dorsal portions of the CNS to provide a subset of myelinating oligodendrocytes [27,46-49], we wanted to determine whether the dorsal glial restricted progenitor cells we identified *in vitro *could participate in the myelination of the forebrain.

**Figure 11:**
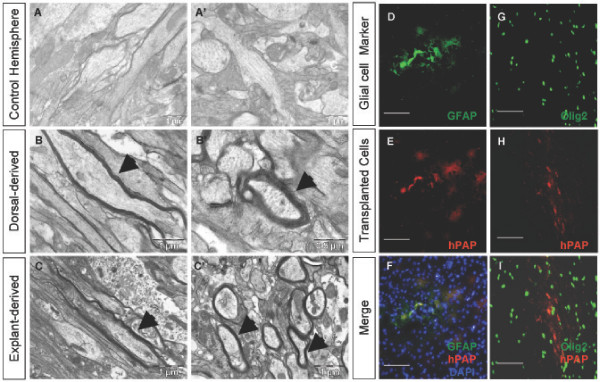
**Dorsal glial restricted progenitors and explant derived dorsal glial restricted progenitors produce compact myelin, in addition to the ability of both ventral and dorsal glial restricted progenitors to make astrocytes *in vivo***. (A-C') EM images from the contralateral hemisphere of the transplanted shiverer forebrains showed a lack of dense, compacted myelin, consistent with the shiverer mutant phenotype, on longitudinally sectioned (A) and cross-sectioned (A') neuronal fibers. The dorsal glial restricted progenitor isolated from the E15 dorsal telencephalon and transplanted into the P18 shiverer forebrain is capable of myelin formation as seen in longitudinally sectioned (B) and cross-sectioned (B') neuronal fibers. Transplantation of the dorsal glial restricted progenitor cell derived from two day *in vitro *grown E13 dorsal telencephalic explants into the P18 shiverer mutant forebrain produces compacted myelin as seen in longitudinally sectioned (C) and cross-sectioned (C') neuronal fibers. (D-F) hPAP+ dorsal glial restricted progenitors transplanted into the forebrains of P0 rat pups generate hPAP+/GFAP+ cells after 10 days, as well as Olig2+ oligodendroglial cells (G-I). DAPI, blue nuclear stain (F). Scale bars for A-C' as indicated, scale bars for D-I, 100 μm.

We isolated A2B5+/PSA-NCAM- glial restricted progenitor cells from 1.) the E15 dorsal telencephalon and 2.) E12.5 dorsal telencephalic explants grown *in vitro *for two days for transplantation into the forebrain of postnatal *shiverer *mice. The *shiverer *mouse contains a deletion in the MBP gene resulting in little to no compacted myelin formation [50-52]. This animal provided an avenue for examining the ability of the dorsal glial restricted progenitor population to generate functional oligodendrocytes that, importantly, can contribute to the myelin composition of the forebrain. The dorsal and explant derived glial restricted progenitor populations were transplanted into the subcortical region of the left hemisphere of postnatal day 18 homozygous *shiverer *mice. The contralateral hemisphere of each mouse was not injected and served as the control for basal myelin presence and appearance. At three weeks post-transplantation, animals were perfused and 1.5 mm coronal sections were prepared for electron microscopy. EM images taken of the non-injected hemispheres showed thin, non-compacted myelin sheets, typical of *shiverer *forebrains, in longitudinally sectioned (Fig. 11A) and cross-sectioned (Fig. 11A') axonal fibers present in the coronal sections. EM images of the hemisphere containing the transplanted E15 dorsal glial restricted progenitor population showed numerous dense, compacted myelinated fibers in the subcortical white matter, seen in longitudinally sectioned fibers (Fig. 11B) and cross-sectioned fibers (Fig. 11B'), extending from the site of injection to more lateral aspects of the dorsal forebrain. Longitudinal and cross-sections of dense, compacted myelinated fibers were readily identifiable in EM images acquired from coronal sections of the hemisphere containing the transplanted explant derived glial restricted progenitor population as well (Figs. 11C and 11C').

One hallmark of the spinal cord derived GRP cell that distinguishes this cell from an OPC is its ability to produce astrocytes upon transplantation [10]. In order to determine the *in vivo *astrocytic potential of the dorsal and ventral telencephalic glial restricted progenitor cells, isolated glial restricted progenitor populations from E15 telencephala of transgenic rat embryos expressing human placental alkaline phosphatase (hPAP; [53]) were transplanted into the forebrains of P0 Sprague Dawley rat pups, a time point coinciding with peak astrocyte formation [54] and the beginning of dorsal born oligodendrocyte precursors [27]. At postnatal day 10, pups were sacrificed and sections were analyzed for co-localization of hPAP and GFAP. Double positive cells could be found throughout the transplanted regions of host brains receiving dorsal (Fig. 11D–F) glial restricted progenitors, although regions showing hPAP+ cells not co-localizing with GFAP were also seen. Olig2+/hPAP+ cells could also be visualized in the transplanted regions, indicating the presence of oligodendrocyte precursors (OPCs) and/or oligodendrocytes (Fig. 11G–I). These transplantation studies confirmed the ability of the dorsal glial restricted progenitor population to generate myelinating oligodendrocytes, as well as the ability of the dorsal glial restricted progenitor population to generate astrocytes and cells of the oligodendrocyte lineage upon transplantation.

## Discussion

The identification of these newly characterized tGRP cells allows a refinement and a possible unification of the current view of oligodendrocyte generation. The original model for oligodendrocyte generation in the telencephalon consisted of ventral OPC generation with subsequent dispersal throughout the CNS [21,46,55-57]. More recent evidence both corroborates this original idea and necessitates its modification to include multiple sites of oligodendrocyte generation, implicating regions of both the ventral and dorsal CNS [22,23,48,58]. In addition to the regional aspects of the origins of OPC generation, there is also a temporal facet to the generation of OPCs. Three waves of oligodendrocyte precursor formation have been described: OPCs are thought to first originate from a region consisting of the *Nkx2.1 *expressing anterior entopeduncular area and medial ganglionic eminence [31]. A second wave of OPC generation is thought to originate from the *Gsh2 *expressing domain of the lateral and/or caudal ganglionic eminence, and the third wave is believed to initiate from the dorsal *Emx1 *expressing domain [27].

The aspects of regional distribution of OPCs together with the timing of oligodendrocyte generation *in vivo *led to a relatively new model that argues for competing waves of telencephalic oligodendrocyte formation, with a "turf war" between the differing OPC populations resulting in a loss of a ventral population and the final oligodendrocyte population being derived from the remaining cell pools [27]. This model is based on observations using separate Cre-*lox *transgenic animals, with *Cre *expression driven by a specifically chosen transcription factor unique to the individual OPC populations, crossed to a *Rosa26R-GFP *transgenic reporter line. Yue et al., 2006 further substantiated the role of dorsally derived OPCs using a cortical specific Olig2 ablation strategy. In spatially restricted ablated animals, the loss of any dorsally derived OPCs created an insurmountable myelin deficit, even with ventral OPC infiltration. These data, however, are in opposition to a recent publication in a separate study. Nakahira et al. (2006) used a Cre-*lox *system to drive *EGFP *expression in a non-cell-specific manner, and attributed at least a subset of dorsal oligodendrocytes to a migrating ventral OPC population [49].

We believe that one of the major obstacles in unifying these observations is a lack of knowledge regarding the cells from which OPCs are generated. In all of the studies mentioned, the cell types targeted are the direct precursors of the myelin forming oligodendrocytes, the OPCs, with little to no attention given to understanding intermediate cell types involved in the generation of the OPCs. In addition, none of the models address a possible source of astrocytes, the major glial component of the CNS. Our studies now provide for the first time the characterization of a cell population that is generated before or in addition to OPCs and could be the possible cell source for dorsal and/or ventral OPC generation, independent of ventral to dorsal cell migration.

Importantly, the identification of tGRPs also offers for the first time a defined potential source for astrocytes. It has been shown in the spinal cord that astrocytes occur in both dorsal and ventral regions [59], and a subset of astrocytes and oligodendrocytes is shown to arise from cells of ventral origin migrating to and residing in the dorsolateral subventricular zone [24,28]. Astrocytic populations have also been identified in other regions of the developing telencephalon [28,32,60-62], but the source of these cells has remained elusive. tGRPs that arise both ventrally and dorsally could account for the generation of at least a subset of astrocytes in the developing telencephalon.

The identification of GRP cells in both spinal cord and telencephalon begs the question of whether the process of gliogenesis involves similar cellular components independent of where in the CNS mature glial cells are produced. The models that are discussed for gliogenesis are to date specific for spinal cord versus telencephalon and include (i) the motorneuron-oligodendrocyte precursor model (MNOP) for spinal cord, (ii) a possible neuron-oligo model specific to the telencephalon [24,26], (iii) a sequential model [63] for spinal cord gliogenesis and (iv) the glial restricted progenitor/neuron restricted progenitor (GRP/NRP) model that was confined to the spinal cord due to the lack of comparable cell types being identified in the telencephalon. The GRP/NRP model is currently the model most supported by *in vitro *as well as *in vivo *studies. Spinal cord GRP cells, shown to be generated from more primitive NSC cells [39], are likely to represent the source for both OPCs and astrocytes in the spinal cord. With the identification of the tGRP populations from regions where OPCs and astrocytes have been described to appear, it is plausible that a similar paradigm exists in the embryonic telencephalon.

**Figure 12:**
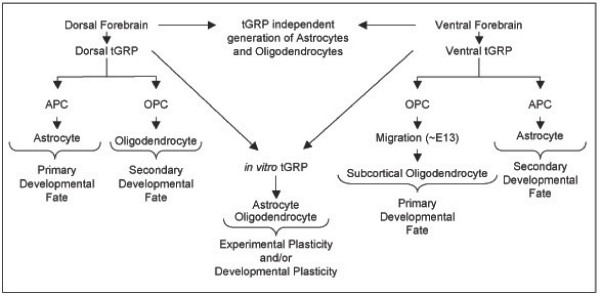
**Proposed model for telencephalic Glial Restricted Progenitor (tGRP) dependent generation of glial subtypes**. The dorsal telencephalon and ventral telencephalon give rise to glial restricted progenitor populations with a primary developmental fate towards astrocyte and OPC generation, respectively. The classification of these two populations as true tGRP populations requires their isolation and *in vitro *characterization in order to remove the normal developmental cues promoting dorsal astrocyte generation and ventral OPC formation. As the ventral and dorsal telencephalon continues through development, each tGRP population has the potential to participate in a secondary developmental fate towards astrocytes ventrally, or OPCs dorsally. The developmental plasticity of each population is revealed *in vitro *and demonstrates the potential for oligodendrocyte and astrocyte development from a common precursor cell type. tGRP independent generation of glial subtypes is represented as well.

The identification of tGRPs allows for the unification of the various existing models of glial origin, and to this end we propose the following model for tGRP dependent gliogenesis in the telencephalon (Figure 12). Our data show that at least two tGRP populations are generated independently in the ventral and dorsal aspect of the embryonic telencephalon. We propose that the dorsal tGRP population is developmentally fated towards APC and astrocyte generation early in development, while the ventral tGRP population shows an initial developmental fate towards OPC generation due to environmental signals. Removal of environmental cues (e.g. BMP dorsally and Shh ventrally) by isolation and *in vitro *culture allows for the emergence of the developmental plasticity of each population, as seen with the generation of astrocytes and oligodendrocytes from ventral and dorsal tGRPs, respectively. Later in development, as signals change or are modified to provide a permissive environment for glial cell maturation, this model affords the potential of each tGRP population to contribute to the generation of an alternate glial cell type, revealing the secondary developmental fate of each tGRP population. Notably, the isolation of a prototypical tGRP population from either the ventral or dorsal regions, regardless of the time point, would provide a cell population capable of generating both oligodendrocytes and astrocytes, but not neurons. This model could account for the complexity of glial generation and the various *in vivo *observations using genetic models that are associated with oligodendrocyte generation. Importantly, this model also addresses the potential source of astrocytes, an aspect of gliogenesis that is largely unexplored and not accounted for in other existing models.

## Conclusion

In the current study, we identified two A2B5+/PSA-NCAM- cell populations: one isolated from the E15 dorsal telencephalon and the other isolated from the E15 ventral telencephalon. An elaborated analysis of the antigenic profile of these cells that included numerous, currently used antigenic markers was useful but remains non-conclusive. For example, the antigenic profile of the A2B5+/PSA-NCAM- (see Table 1) would be consistent with the cells representing either GRP cells or stem cells. The overlap of antigenic markers of GRP cells with neural stem cells, together with the now emerging possibility of multiple origins of OPCs, has made it difficult to distinguish cells from each other and to assign identities to specific cell pools. It seems clear that the designation of cells as GRP, OPC or NSC requires a more detailed and stringent analysis. One of the most reliable tools that distinguishes stem cells from lineage restricted precursor cell pools is the analysis of the cell type-specific differentiation potential (for review, see Noble et al 2006). While it can be expected that NSC will be able to generate oligodendrocytes, astrocytes and neurons, lineage restricted cells would not display the full array of cell types upon differentiation.

The mass culture analyses, clonal analyses, clone splitting analyses, and *in vivo *transplantation experiments of the A2B5+/PSA-NCAM-/beta-III tubulin- telencephalic cell population demonstrated their ability to generate cells of the glial lineage but an inability to differentiate into neurons. This differentiation profile strongly resembles that of the previously described GRP population of the E13.5 spinal cord [7]. In addition to the similar differentiation profile, the telencephalic glial restricted progenitor populations are, like the spinal cord GRP population, responsive to bFGF as a mitogen and survival factor [7] and can also be isolated from both dorsal and ventral aspects of the respective tissues [4]. Our data also establishes the capability of the dorsal telencephalon to generate a telencephalic glial restricted progenitor population in the absence of ventral cell tissue. These observations suggest that the dorsally derived GRP cell population might actually represent the source for at least a subset of dorsally derived mature glial cells.

There were, however, readily detectable differences between the telencephalic cells and the previously studied spinal cord cells, including the astrocyte generation upon exposure to BMP-4, as well as a lack of type-2 astrocyte generation in response to CNTF [7]. The last characteristic, in particular, makes a distinction between this novel progenitor cell population and the extensively studied OPCs isolated from postnatal rat brains. In order to distinguish this telencephalic glial restricted population from not only the PDGFR-alpha+ OPC population, but also the spinal cord GRP population, we suggest the designation of the telencephalic cell populations as telencephalic glial restricted progenitors (tGRP).

Future topics of interest include the characterization of the time point of generation of the tGRP populations in the telencephalon, the contribution of each population to the mature glial cell component in their respective locations, and the potential presence of a natural or inducible fate switch to occur allowing for the modulation of glial cell type generation. As the tGRP populations are present during the peak of neuron formation, a contribution by this population to the cytoarchitectonics of the early telencephalon may also be of considerable interest.

## Methods

### Cell culture

A2B5+/PSA-NCAM- cells were isolated from embryonic day 15 (E15) Sprague Dawley rat telencephala using A2B5 and an antibody recognizing the polysialylated form of neural cell adhesion molecule (PSA-NCAM) [7,39,64] in combination with magnetic separation using Miltenyi MACS Cell Separation Columns (Miltenyi Biotech). For explant studies, the dorsal telencephala was removed from E13 Sprague Dawley rats and placed on Millicell culture plate inserts for two days of *in vitro *growth in Neural Basal Media (Gibco) with the addition of 2 mM Glutamax (Gibco) and B27 Supplement minus AO (Gibco), before being immunopurified as above. Cells were grown on fibronectin/laminin-coated glass coverslips at 1000 cells per well of a 24 well plate for mass culture experiments or at 500 cells per T25 flask and/or 40 cells per well of a 24 well plate for clonal analysis. For propagation, cultures were grown in DMEM-F12 supplemented with additives described by Bottenstein and Sato [65] and basic fibroblast growth factor (bFGF: 10 ng/ml). At the specified time, cells were stained with A2B5 antibody [66] to detect precursor cells, anti-galactocerebroside (GalC) [67] to identify oligodendrocytes, anti-GFAP antiserum to identify astrocytes [68,69] and anti-beta-III tubulin [70] to detect neurons, followed by the appropriate fluorochrome conjugated secondary antibodies (Molecular Probes).

### *In vitro *culture of A2B5+/PSA-NCAM- cells and the loss of beta-III tubulin+ cells

Over the course of a one week *in vitro *culture period, the cells were passaged twice, which resulted in a loss of the A2B5+/PSA-NCAM-/beta-III tubulin+ neuronal population (see Figure 3C). The loss of this neuronal population is attributable to two factors: (i) our medium condition was not permissive for the survival of the neuronal A2B5+/PSA-NCAM-/beta-III tubulin+ cells, but was sufficient to allow survival and proliferation of the non-neuronal A2B5+/PSA-NCAM-/beta-III tubulin- population, and (ii) a difference in substrate binding between the neuronal and putative glial progenitor populations. To attribute the loss of the neuronal population to the growth conditions used for expansion of the putative GRPs, we also cultured the freshly isolated, heterogeneous A2B5+/PSA-NCAM- populations in the presence of PDGF-AA, a factor that has been shown to support neuronal survival [34]. We found that this condition was supportive of the survival of A2B5+/PSA-NCAM-/beta-III tubulin+ cells, with a detection of non-neuronal A2B5+/PSA-NCAM-/beta-III tubulin- cells at no more than two cells per 1 × 10^6 ^total cells (0.0002%; data not shown). In regards to the observed difference in substrate binding, the neuronal A2B5+/PSA-NCAM-/beta-III tubulin+ cells were less adherent to fibronectin/laminin coated surfaces when compared to the A2B5+/PSA-NCAM-/beta-III tubulin- cells and contributed to the loss of the neuronal population during passaging. As a direct application of the above findings, growth of the freshly isolated A2B5+/PSA-NCAM- population (containing both putative glial restricted progenitors and neurons) in 10 ng/ml bFGF on fibronectin/laminin resulted in the purification of the non-neuronal A2B5+/PSA-NCAM-/beta-III tubulin- population.

### Mass culture and clonal analysis of telencephalon populations

Mass culture and clonal differentiation analyses were used to confirm the differentiation potential of cell populations and individual precursor cells, respectively, as used previously in GRP cell characterization from the spinal cord [7,10,64], as well as in characterization of OPCs [71,72]. Cells were isolated as described above and grown in bFGF for 1 week prior to replating for mass culture or clonal density. Cells were propagated in bFGF for 2 days prior to exposure to one of the following conditions: 10 ng/ml bFGF (control: proliferative), 10 ng/ml Bone Morphogenic Protein 4 (BMP-4: astrocyte induction), 2% Fetal Bovine Serum (FBS: astrocyte induction), 1 ng/ml Platelet Derived Growth Factor (PDGF-AA) plus a mixture of 49 nM Triiodothyronine and 45 nM Thyroxine (PDGF-AA + T3/T4: oligodendrocyte induction), or 10 ng/ml Neurotrophin-3 plus 100 nM Retinoic Acid (NT3 + RA: neuron induction).

### Section preparation

Embryos from various developmental ages were immersed in cold isopentane (Sigma) and stored at -80°C until sectioned. 10 μm sections were cut using a Shandon Cryotome Cryostat and collected on Superfrost Plus slides (VWR). Slides were air dried at room temperature overnight and processed for primary antibody staining or stored at -80°C. Sections were fixed by immersion in 4% paraformaldehyde for 10 minutes at room temperature followed by a 2 minute acetone exposure at -20°C. All washing steps were carried out in Tris buffered saline. Blocking buffer consisted of 0.5 M TBS with 5% Goat Serum and 4% Bovine Serum Albumin.

### Fluorescence activated cell sorting analysis

Freshly dissociated cells were stained with primary antibodies that included anti-PSA-NCAM with a secondary anti-IgM-PE conjugate, and A2B5 conjugated directly to fluorescein. FACS staining was conducted at 4°C in the following sequence: Primary PSA-NCAM, secondary IgM-PE, primary A2B5-FITC. Flow cytometry was performed on a Becton Dickinson FACSCalibur and analysis was done using CELLQuest software.

### Immunostaining of cells and sections

All primary antibody stains were done at 4°C overnight, followed by a 30 minute stain with the appropriate secondary. A2B5, PSA-NCAM, O4, Ran2 and GalC hybridoma supernatants (American Type Culture Collection) were used at 1:10 dilutions. 3CB2 and RC2 hybridoma supernatants (Developmental Studies Hybridoma Bank) were used at 1:50. GFAP rabbit polyclonal antibody (Dako) and beta-III tubulin (BioGenex) were used at 1:400. Sox2 (Chemicon), Sox10 (Sigma), Nestin (Rat 401; Chemicon), NG2 (Chemicon), S100 (Chemicon) and PDGFR alpha (Santa Cruz Biotechnology) antibodies were used at 1:500. CD44 antibody (Accurate) and human Placental Alkaline Phosphatase antibody (Sigma) were used at 1:1000. An Olig2 specific polyclonal antibody [25,73,74] (a generous gift from D. Rowitch) was used at 1:40,000. All secondary antibodies were purchased from Molecular Probes and included goat anti-mouse IgG3, IgM, IgG2a, and goat anti-rabbit Ig (heavy and light chain) conjugated to Alexa-488, Alexa-350, Alexa-546 or Alexa-568.

### Clonal splitting experiments

Immunopurified cells were plated at clonal density and grown in 10 ng/ml bFGF until clones were detected containing approximately 200 cells. These clones were then selectively passaged and split into four separate wells containing one of the following: 10 ng/ml bFGF, 2% FBS, 1 ng/ml PDGF-AA plus a mix of 45 nM T3 and 49 nM T4, or 10 ng/ml NT-3 plus 100 nM RA. Media was changed every other day for six days and cells were processed for immunostaining as indicated above.

### Transplantation

Postnatal day 18 homozygous shiverer mice were anesthetized with 25 μl of a 100 μg/μl solution of ketamine prior to transplantation. A 0.34 mm needle was used to inject 1.5 μl of PBS containing 1 × 10^5 ^A2B5+/PSA-NCAM- cells at four injection sites lateral to the cortical hem of the left hemisphere. The needle was inserted to a depth of 3 mm and remained in the injection site for 1 minute prior to removal. *Shiverer *mice undergoing the transplantation procedure were sacrificed three weeks post-transplantation for analysis. Postnatal day 0 Sprague Dawley rat pups were anesthetized by hypothermia for hPAP expressing, telencephalic cell transplantation. 8–9 sites were injected with 27.6 nl per injection site at a depth of 1 mm into the left hemisphere. Rat pups receiving cell transplantations were sacrificed at postnatal day 10 and processed for immunofluorescence as described above.

### Electron microscopy

Animals that underwent cell transplantation were perfused with a mixture of paraformaldehyde and gluteraldehyde warmed to 38°C. Brains were removed and sectioned into 1 mm coronal sections using a Braintree Scientific 1 mm mouse acrylic matrix. Each section was fixed overnight in paraformaldehyde/gluteraldehyde mix, rinsed with phosphate buffer, pH 7.4, and post-fixed in phosphate buffered 1.0% osmium tetroxide for 1.5 hours. The 1 mm sections were dehydrated in a graded series of ethanol (ETOH) to 100%, transitioned into 100% propylene oxide and infiltrated in Epon/Araldite (Electron Microscopy Sciences, Fort Washington, PA) epoxy resin overnight. Sections were embedded into molds with fresh resin and polymerized for two days at 70°C. Semi-thin two micron sections were cut and stained with 0.5% toluidine blue in 2% sodium borate and examined under a light microscope to determine the area to be thin sectioned. Thin sections were cut with a diamond knife and placed on 200 mesh copper grids and stained with uranyl acetate and lead citrate. The grids were examined with a Hitachi 7100 Transmission Electron Microscope and digital images were captured using a MegaView III digital camera (AnalySIS, Lakewood, CO).

## Authors' contributions

FS carried out the dissections, cell culture, mass and clonal analyses, section preparation, FACS analyses, immunostaining of cells and sections, and clone splitting experiments. XW conducted the shiverer and P0 transplantations, MMP conceived of the study, and participated in its design and coordination and helped to draft the manuscript. All authors read and approved the final manuscript.
